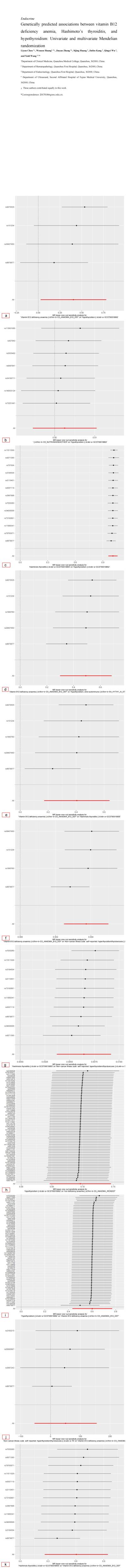# Unveiling the clinical connections between vitamin B12 deficiency anemia, Hashimoto thyroiditis, and hypothyroidism: New insights from Mendelian randomization studies

**DOI:** 10.1097/MD.0000000000048901

**Published:** 2026-05-15

**Authors:** Liyun Chen, Wensen Huang, Jinyan Zhang, Sijing Huang, Jinfen Kang, Qingyi Wu, Xiali Wang

**Affiliations:** a Department of Clinical Medicine, Quanzhou Medical College, Quanzhou, Fujian Province, China; b Department of Endocrinology, Quanzhou First Hospital, Quanzhou, Fujian Province, China; c Department of Hematopathology, Quanzhou First Hospital, Quanzhou, Fujian Province, China; d Department of Ultrasound, Second Affiliated Hospital of Fujian Medical University, Quanzhou, Fujian Province, China.

**Keywords:** B12DA, Hashimoto thyroiditis, hypothyroidism, iron deficiency anemia, thyrotoxicosis, univariate and multivariate Mendelian randomization

## Abstract

Clinical evidence indicates a strong association between nutritional anemia and hypothyroidism. However, whether nutritional factors contribute directly to the onset of hypothyroidism remains unclear. In this study, we aimed to address this knowledge gap by examining the causal relationship between nutritional anemia and hypothyroidism. We conducted rigorous univariate and multivariate Mendelian randomization (MVMR) analyses using data from the Integrative Epidemiology Unit Open Genome-wide Association Studies database. We found that both vitamin B_12_ deficiency anemia (B12DA) and iron deficiency anemia (IDA) have a positive influence on hypothyroidism. However, whereas MVMR analysis revealed that the causal relationship between B12DA and hypothyroidism persisted after adjusting for IDA, no significant causal associations were observed between IDA (adjusted for B12DA) and hypothyroidism. In addition, we detected a causal relationship between Hashimoto thyroiditis and hypothyroidism, and that B12DA had a significant causal effect on Hashimoto thyroiditis. MVMR analysis further revealed that after adjusting for each other, the causal relationships among B12DA, Hashimoto thyroiditis, and hypothyroidism persisted. Finally, although B12DA and Hashimoto thyroiditis are both risk factors for thyrotoxicosis, the relationship between B12DA and thyrotoxicosis disappeared after adjusting for Hashimoto thyroiditis in MVMR analysis. Our findings indicate the role of B12DA in hypothyroidism development and that Hashimoto thyroiditis mediates the causal pathway between B12DA and hypothyroidism. These findings emphasize the importance of considering nutritional status, particularly B_12_ deficiency, in the context of hypothyroidism and anemia, and potentially provide new insights for their management.

## 1. Introduction

Hypothyroidism, a condition characterized by an insufficient production of thyroid hormones, can have a range of harmful effects on the body. Anemia is a recognized complication associated with hypothyroidism. Ali et al identified iron deficiency anemia (IDA) and vitamin B_12_ deficiency anemia (B12DA) as prevalent types of anemia.^[1]^ However, although nutritional anemia, including IDA and B12DA, often occurs concomitantly with hypothyroidism,^[2]^ most relevant studies have mainly focused on the effects of thyroid disorders on anemia, whereas research on the impact of anemia on thyroid function remains limited.

The prognosis and potential complications associated with anemia vary significantly depending on the type of anemia. Consequently, it is necessary to identify the type of anemia that leads to hypothyroidism to develop more targeted and effective treatments.^[1]^ In this regard, multivariate Mendelian randomization (MVMR), a powerful tool for disentangling the independent and joint effects of multiple exposures on an outcome,^[3]^ can provide insights into the complex causal relationships between B12DA and hypothyroidism. Under circumstances in which secondary exposure serves as a mediator in the relationship between the primary exposure and outcome, MVMR can yield a consistent estimate of the direct impact of B12DA on hypothyroidism, independent of the mediating pathway.^[4]^

Hashimoto thyroiditis, the most common cause of hypothyroidism, ranks among the most prevalent autoimmune thyroid diseases globally,^[5]^ and its prevalence is currently rising. Given the potential moderating role of Hashimoto thyroiditis in the causal link between vitamin B_12_ deficiency and hypothyroidism, gaining a more comprehensive understanding of this relationship is of considerable clinical relevance for accurately diagnosing, managing, and developing preventive strategies for these disorders.

Although a previous Mendelian randomization (MR) study has provided evidence of a causal relationship between IDA and hypothyroidism,^[6]^ the causal relationship between hypothyroidism and B12DA or other types of nutritional anemia remains unclear. MR analysis can be performed in parallel with randomized controlled trials, utilizing genetic variants as instrumental variables (IVs) to evaluate the causal association between 2 variables. This approach contributes to eliminating confounding factors and reverse causation biases that are often encountered in observational research.^[7]^ In this study, we used MR analysis to investigate whether anemia acts as a pathogenic factor in hypothyroidism.

## 2. Methods

### 2.1. Study design

**Figure 1.**
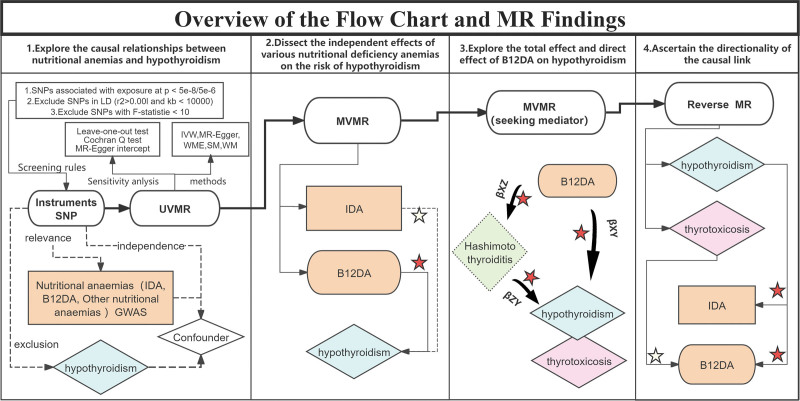
Overview of the flowchart and Mendelian randomization findings. 1. Application of genetic variants as instrumental variables is contingent upon meeting the following 3 key assumptions. Relevance: there must be a strong association between the genetic variants and the exposure. Independence: the genetic variants selected as instrumental variables should not be associated with confounding factors. Exclusion: the genetic variants should influence the outcome exclusively via exposure, with no other pathways involved. 2. Dissection of the independent effects of different nutritional deficiency anemias on the risk of hypothyroidism. 3. The direct effect of B12DA on hypothyroidism refers to the effect of B12DA on hypothyroidism independent of Hashimoto thyroiditis, denoted β*XY*. The total effects of B12DA on hypothyroidism include both direct effects and the indirect effects mediated through Hashimoto thyroiditis, represented as β*XY* + β*XZ*β*ZY*. 4. Ascertaining the directionality of the causal link. A 5-pointed red star indicates a causal relationship, whereas a 5-pointed white star indicates no causal relationship. B12DA = vitamin B_12_ deficiency anemia, IDA = iron deficiency anemia, IVW = inverse variance weighted, MR = Mendelian randomization, MVMR = multivariate Mendelian randomization, SM = simple mode; SNP = singlenucleotide polymorphisms, UVMR = univariate Mendelian randomization, WM = weighted mode, WME = weighted median estimator.

This study was preregistered on the Open Science Framework (OSF) (Registration ID: osf.io/2edu6). This study used publicly available summary-level Genome-wide Association Studies (GWAS) datasets and did not involve the collection of new individual-level data. The original GWAS studies had been approved by the relevant ethics committees or institutional review boards, as reported in the original studies and database records, and informed consent had been obtained from the participants. The research protocol, including the definitions of exposure and outcome, the selection of instrumental variables, and the analytical approaches, was established prior to data analysis and is comprehensively detailed in the Methods section. Utilizing a 2-sample Mendelian MR framework, we aimed to delineate the causal relationships between the 3 subtypes of nutritional anemia (iron deficiency anemia, B12DA, and other nutritional anemias) and the onset of hypothyroidism. In the analyses, B12DA was treated as a binary variable (deficient vs nondeficient), while hypothyroidism was defined based on clinical diagnosis. Genetic associations were scaled per standard deviation increase in genetically predicted B12DA risk. Subsequently, a MVMR approach was employed to determine the independent effects of different nutrient deficiency anemias on the risk of hypothyroidism. Univariate Mendelian randomization and MVMR methods were used to examine the mediating role of Hashimoto thyroiditis in the causal effects of B12DA and hypothyroidism. The relationships among B12DA, Hashimoto thyroiditis, and hypothyroidism are illustrated in Figure 1. The direct effect of B12DA on hypothyroidism is the effect that B12DA has on hypothyroidism independent of Hashimoto thyroiditis (β*XY*). The total effect of B12DA on hypothyroidism is the direct effect of B12DA on hypothyroidism plus the effect of B12DA on hypothyroidism via Hashimoto thyroiditis (β*XY* + β*XZ*β*ZY*). Reverse MR was applied to ascertain the directionality of the causal link between anemia and hypothyroidism (Fig. 1).

### 2.2. GWAS database

Genetic association data for the selected variants with the exposures and outcomes were sourced from the Integrative Epidemiology Unit Open GWAS database (https://gwas.mrcieu.ac.uk/). Details of the included studies are provided in Table 1. To mitigate the risk of population stratification biases, all participants with anemia and hypothyroidism were European. The GWAS data on IDA were derived from a study published by FinnGen (ID: finn-b-D3_ANAEMIA_IRONDEF) in 2021, which included 6087 Europeans (3905 females and 2182 males). Similarly, the GWAS data on B12DA were obtained from another FinnGen study (ID: finn-b-D3_ANAEMIA_B12_DEF) published in the same year, involving 1707 Europeans (952 females and 755 males). Additionally, the GWAS data on hypothyroidism were sourced from a study by Sakaue et al (ID: ebi-a-GCST90018862) in 2021, comprising 30,155 European ancestry cases, 379,986 European ancestry controls, 1114 East Asian ancestry cases, and 172,656 East Asian ancestry controls. In the same publication, the GWAS data on Hashimoto thyroiditis were also provided by Sakaue et al (ID: ebi-a-GCST90018855), with a cohort of 15,654 European ancestry cases, 379,986 European ancestry controls, 537 East Asian ancestry cases, and 172,656 East Asian ancestry controls. These studies collectively provide valuable genetic insights into IDA, B12DA, hypothyroidism, and Hashimoto thyroiditis across diverse populations. The diagnosis of IDA relies on International Classification of Diseases (ICD-10) codes (D50), laboratory test results (e.g., low hemoglobin and ferritin levels), and clinical data. Similarly, the diagnosis of B12DA is based on ICD-10 codes (D51), laboratory findings (e.g., low vitamin B12 levels, elevated methylmalonic acid, and homocysteine), and clinical data. For hypothyroidism, the diagnosis is established using ICD-10 codes (E03), laboratory test results (e.g., elevated TSH and decreased FT4), and clinical data. Additionally, the diagnosis of Hashimoto thyroiditis is determined by ICD-10 codes (E06.3), laboratory results (e.g., elevated thyroid peroxidase antibody and thyroglobulin antibody), imaging studies (e.g., thyroid ultrasound), and clinical data. These comprehensive diagnostic criteria ensure accurate identification and analysis of IDA, B12DA, hypothyroidism, and Hashimoto thyroiditis.^[8]^

**Table 1:** Overview of data acquired from Genome-wide Association Studies database.

Variable name	Year	Population	Number of cases	Number of SNPs	GWAS ID
IDA	2021	European	6087	16,380,461	finn-b-D3_ANAEMIA_IRONDEF
B12DA	2021	European	1707	16,380,452	finn-b-D3_ANAEMIA_B12_DEF
Other nutritional anemia	2021	European	145	16,380,446	finn-b-D3_NUTRIANAEMIAOTHER
Hypothyroidism	2021	European	30,155	24,138,872	ebi-a-GCST90018862
Thyrotoxicosis	2017	European	16,376	10,894,596	ukb-a-76
Hashimoto thyroiditis	2021	European	15,654	24,146,037	ebi-a-GCST90018855

B12DA = vitamin B12 deficiency anemia, GWAS = Genome-wide Association Studies, IDA = iron deficiency anemia, SNPs = single nucleotide polymorphisms.

### 2.3. Genetic IVs

The selected genetic variants serving as IVs were assessed to ensure a strong correlation with the exposures and no links with confounding factors. They adhered to the 3 key assumptions of MR: relevance, independence, and exclusion (Fig. 1). IVs were selected following a systematic process. First, association analysis involved selecting single nucleotide polymorphisms (SNPs) showing a strong correlation with the exposure, using a significance threshold of *P *<* *5*e*^−8^/*P* <* *5*e*^−6^. Second, SNPs in linkage disequilibrium were removed, with the parameters being adjusted to *r*^2^* = *0.001 and a kb (linkage disequilibrium block) of 10,000. Third, weak IVs were excluded by selecting SNPs with an *F* test value >10 for further analysis. Fourth, SNPs with incompatible alleles or palindromic sequences were excluded. Fifth, SNPs associated with confounding factors were removed in reference to online data from the National Institutes of Health (https://ldlink.nih.gov).

### 2.4. MR analysis

MR analysis was conducted using R software (version 4.4.1; R Foundation for Statistical Computing, Vienna, Austria), the computer code for which is available from the corresponding author on reasonable request. We primarily utilized the inverse variance weighted (IVW) approach to assess the causal effects of anemia on hypothyroidism.^[9]^ Additional single-instrument methods, such as MR-Egger regression, the weighted median estimator, and both simple and weighted models, were employed to enhance the reliability of our results. The analysis findings indicate that a *P* value of <.05 is taken as evidence of a causal link between the exposure and the outcome. A positive value for beta is considered a risk factor, whereas a negative value for beta is deemed a protective factor. The MR-Polygenic Risk Estimation for SNPs with Systematic Outlier detection technique was applied to detect and remove outlier SNPs,^[10]^ whereas the leave-one-out procedure was used to gauge the impact of individual SNPs on the outcome.^[11]^ Heterogeneity and horizontal pleiotropy were examined using the Cochran *Q* and MR-Egger intercept tests, respectively.

## 3. Results

### 3.1. MR analysis

**Figure 2.**
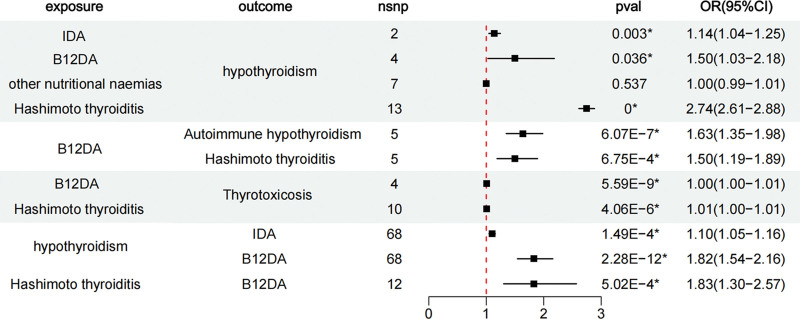
Forest plots of univariate Mendelian randomization analysis. Dots and lines indicate causal estimates of exposure to the risk of outcome. B12DA = vitamin B12 deficiency anemia, CI = confidence interval, IDA = iron deficiency anemia, nsnp = nonsynonymous single nucleotide polymorphism, OR = odds ratio, pval = *P* value.

Causal effects of B12DA and IDA on hypothyroidism: After removing rs1990760 (refer to the National Institutes of Health: https://ldlink.nih.gov), the IVW method revealed a causal relationship between B12DA and hypothyroidism (IVW: odds ratio [OR] = 1.50, 95% confidence interval [CI] = 1.03–2.18, *P *= .036) (Fig. 2). The results obtained using the other 4 methods of analysis were consistent: MR-Egger (OR = 3.81, 95% CI = 2.23–6.54, *P *= .040); weighted median estimator (OR = 1.30, 95% CI = 1.17–1.46, *P *= 3.75E−06); simple model (OR = 1.53, 95% CI = 1.29–1.82, *P *= .017); and weighted model (OR = 1.41, 95% CI = 1.11–1.79, *P *= .068) (Table S1). A similar trend was found between IDA and hypothyroidism (IVW: OR = 1.14, 95% CI = 1.04–1.25, *P *= .003) (Fig. 2). The IVW approach indicated no causal link between other nutritional anemias and hypothyroidism (OR = 1.00, 95% CI = 0.99–1.01, *P *= .537) (Fig. 2).

**Figure 3.**
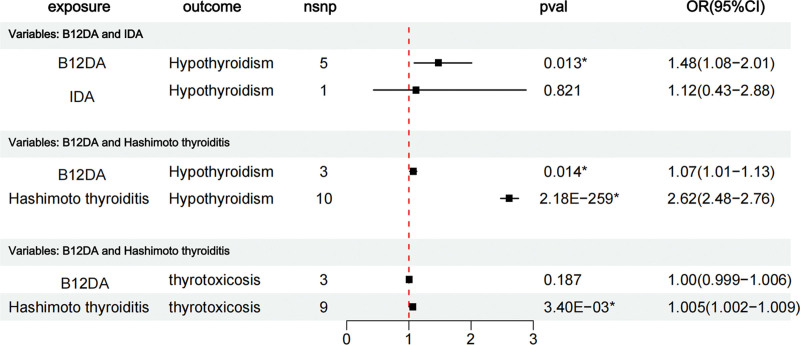
Results of multivariate Mendelian randomization analysis. *P < .05. B12DA = vitamin B12 deficiency anemia, CI = confidence interval, IDA = iron deficiency anemia, nsnp = nonsynonymous single nucleotide polymorphism, OR = odds ratio, pval = *P* value.

MVMR analysis revealed that the causal relationship between B12DA and hypothyroidism (OR = 1.48, 95% CI = 1.08–2.01, *P *= .013) persisted even after adjusting for IDA. However, no significant causal associations were observed between IDA and hypothyroidism (adjusted for B12DA: OR = 1.12, 95% CI = 0.43–2.88, *P* = .821) (Fig. 3).

Hashimoto thyroiditis as a mediator in the causal pathway between B12DA and hypothyroidism: univariate Mendelian randomization analysis indicated a causal effect between B12DA and autoimmune hypothyroidism (IVW: OR = 1.63, 95% CI = 1.35–1.98, *P *= 6.07E−07) (Fig. 2). A causal relationship between B12DA and Hashimoto thyroiditis was also observed (IVW: OR = 1.50, 95% CI = 1.19–1.89, *P *= 6.75E−04) (Fig. 2). Hashimoto thyroiditis was found to have further significant causal effects on hypothyroidism (IVW: OR = 2.74, 95% CI = 2.61–2.88, *P *= .000) and weak causal effects on hyperthyroidism (IVW: OR = 1.01, 95% CI = 1.00–1.01, *P *= 5.59E−09).

MVMR analysis further showed that after adjusting for Hashimoto thyroiditis, the causal relationship between B12DA and hypothyroidism persisted (OR_MVMR_ = 1.07, 95% CI = 1.01–1.13, *P*_MVMR_ = .014), whereas the causal link between B12DA and thyrotoxicosis was precluded (OR_MVMR_ = 1.00, 95% CI = 1.00–1.01, *P*_MVMR_ = .187). The direct effect of B12DA on hypothyroidism is the effect that B12DA has on hypothyroidism independent of Hashimoto thyroiditis, denoted as β*XY* (β*XY* = 0.404, Table S1). The total effect of B12DA on hypothyroidism includes the direct effect of B12DA on hypothyroidism and the indirect effect mediated via Hashimoto thyroiditis, expressed as β*XY* + β*XZ*β*ZY* (β*XY* + β*XZ*β*Z* = 0.813). Additionally, the direct and total effects of B12DA on thyrotoxicosis were 0.004 and 0.006, respectively (Table S1).

Reverse MR analysis: Results obtained using the 5 assessed analytical methods were consistent, thereby providing evidence to indicate that hypothyroidism is a risk factor for B12DA (IVW: OR = 1.82, 95% CI = 1.54–2.16, *P *= 2.28E−12) and IDA (IVW: OR = 1.10, 95% CI = 1.05–1.16, *P *= 1.49E−04). This causal relationship was also observed with Hashimoto thyroiditis (IVW: OR = 1.83, 95% CI = 1.30–2.57, *P *= 5.02E−04), although not with thyrotoxicosis and B12DA (IVW: OR = 7.75E+21, 95% CI = 0.00–1.60E+67, *P *= .344).

### 3.2. Sensitivity analysis in MR

Sensitivity analyses are essential in MR studies for assessing the robustness of causal estimates and to examine the potential for bias caused by violations of the key assumptions underlying the MR approach.

Leave-one-out analysis: The leave-one-out analysis revealed that excluding each genetic variant did not significantly alter the causal estimate, thereby indicating robustness of the inclusion of individual instruments (Figure S1).

Cochran *Q* test: The outcomes of Cochran *Q* test among the different sets of MR analyses revealed differing levels of heterogeneity (Table S2). In the MR analysis examining the causal effect on hypothyroidism and Hashimoto thyroiditis, the findings of Cochran *Q* test indicated significant heterogeneity among the included studies, with a highly significant *P* value of <.05. In contrast, Cochran *Q* test indicated no significant heterogeneities among the included studies, with a *P* value >.05.

MR-Egger intercept test: The MR-Egger intercept test for the association between Hashimoto thyroiditis and hypothyroidism (intercept = −0.022, *P *= .040) revealed a significant directional bias in the estimates. Other MR-Egger regression intercepts did not differ significantly from zero, thereby providing reassurance against substantial bias caused by horizontal pleiotropy (Table S2).

## 4. Discussion

In this study, we used a 2-sample randomization-based MR analysis to investigate the causal relationship between nutritional anemias and hypothyroidism. Our findings revealed that both IDA and B12DA have positive effects on hypothyroidism. Given that the different nutrients that cause anemia often have common sources and can influence 1 another, it is possible that different types of nutritional anemia will coexist in the same individual. Consequently, the effect of different risk factors on the outcome may be confounded by interactions among these different types of anemia. To address this issue, we performed an MVMR analysis to account for the causal influence of different types of nutritional deficiency anemia on hypothyroidism. We incorporated both IDA and B12DA into a single model, with the results of MVMR analysis indicating that after adjusting for IDA, the causal relationship between B12DA and hypothyroidism remained significant. Contrastingly, after adjusting for B12DA, a causal relationship between IDA and hypothyroidism was no longer observed. In studies examining the causal relationship between IDA and hypothyroidism, a notable shift in outcomes has been observed when transitioning from univariate to multivariate MR analysis, with this discrepancy being largely attributable to the effects of B12DA. IDA and B12DA have common risk factors, which include restrictive diets, inadequate nutrient intake, and chronic illnesses. Additionally, these 2 forms of anemia can have reciprocal impacts whereby IDA may hinder the absorption and metabolism of vitamin B_12_, leading to B_12_ deficiency, and B_12_ deficiency can, in turn, impair iron absorption and utilization, thereby exacerbating IDA.^[12]^

We established that B12DA may be the primary pathogenic factor contributing to the development of hypothyroidism, thereby indicating the presence of a biological pathway via which anemia may directly lead to the development of hypothyroidism. Potential mechanisms linking B12DA to hypothyroidism may include impaired DNA synthesis attributable to vitamin B_12_ deficiency,^[13]^ induction of oxidative stress,^[14]^ inflammatory processes secondary to anemia,^[15]^ or broader systemic effects stemming from anemia.^[16]^ However, our genetic findings suggest a more specific biological framework centered on autoimmune susceptibility, rather than mechanisms solely related to the downstream consequences of anemia. In screening for SNPs associated with hypothyroidism among individuals with B12DA, we identified 5 SNPs linked to the condition. Among these, previous studies have reported an association between the rs1990760 polymorphism and autoimmune thyroid disease.^[17]^ To assess whether this single variant drove the observed association, we initially removed rs1990760 and repeated the MR analysis. The positive causal relationship between B12DA and hypothyroidism remained, suggesting that the association was not attributable solely to rs1990760. Previous studies have also linked rs1990760 to interferon induced with helicase C domain 1, a gene involved in antiviral innate immune signaling and autoimmune susceptibility.^[18,19]^ In addition, rs151234 may influence the expression or function of interleukin 27, thereby potentially affecting immune responses, metabolic regulation,^[18]^ and blood cell counts and indices.^[20]^ Another relevant variant, rs6679677, is in linkage disequilibrium with rs2476601, a missense variant (R620W) in protein tyrosine phosphatase nonreceptor type 22, a well-established autoimmune susceptibility locus.^[21]^

Taken together, these findings suggest that the causal relationship between B12DA and hypothyroidism may be partly associated with genetic variation in or near immune-related loci, including interferon induced with helicase C domain 1, interleukin 27, and protein tyrosine phosphatase, nonreceptor type 22. We therefore propose that genetic predisposition involving these autoimmune-related loci may promote a state of generalized autoimmune dysregulation. This shared immune predisposition may contribute to both autoimmune-mediated vitamin B12 deficiency, such as pernicious anemia or autoimmune gastritis, and autoimmune thyroid disease, which may ultimately manifest as hypothyroidism.

Under this framework, the relationship between B12DA and hypothyroidism should not be interpreted simply as a linear pathway in which nutritional deficiency directly causes thyroid dysfunction. Instead, these 2 conditions may partly share a common upstream autoimmune diathesis. Once B12DA develops, it may further aggravate immune dysfunction through altered immune-cell activity, impaired redox homeostasis, or epigenetic changes, thereby potentially contributing to the progression or clinical expression of thyroid dysfunction. This integrated framework better reconciles the autoimmune genetic evidence with our MR findings, including the mediation and bidirectional analyses.

In this study, we performed a bidirectional MR to examine the causal relationship between the 2 diseases, the results of which revealed that B12DA contributes to the onset of hypothyroidism, and conversely, hypothyroidism also exacerbates B12DA development. This bidirectional relationship highlights the certain limitations in using traditional observational studies to investigate the effects of B12DA on thyroid function. In contrast, MR analysis is unaffected by reverse causality, which is one of the strengths of this procedure. To the best of our knowledge, this study is the 1st to use MR methodology to provide evidence that B12DA contributes to the development of hypothyroidism. The bidirectional relationship between these 2 conditions reveals the complex interplay between nutritional status and thyroid function, thus emphasizing the importance of integrated management strategies that address the nutritional and endocrine aspects of these interconnected diseases.

These findings are consistent with those reported by Huang et al,^[6]^ who employed MR analysis to identify an association between IDA and hypothyroidism in a European population. Specifically, these authors focused on the relationships among iron status, thyroid dysfunction, and IDA. In the present study, we focused on the effects of anemia on the associations of deficiencies in iron, vitamin B_12_, and other nutrients with hypothyroidism. This accordingly highlights the interconnectedness of diverse nutrient deficiencies, particularly the interplay between iron and vitamin B_12_, which may have implications for hypothyroidism. The coexistence of different nutrient deficiencies may contribute to complicating determinations of the independent effects of a single nutrient deficiency on hypothyroidism in traditional randomized controlled trials. Our application of MVMR analysis to mitigate the potential confounding effects of the interrelationships among different nutrient deficiencies can thus be considered an innovative approach. Consequently, when a case of anemia is identified, it is important to consider the potential occurrence of different types of anemia and develop a treatment strategy that combines iron and vitamin B_12_ supplementation, which is essential for the effective clinical management of hypothyroidism.

Given that autoimmune thyroid disease, particularly Hashimoto thyroiditis, is the most common cause of hypothyroidism,^[22]^ we used a 2-sample MR approach to investigate the genetic association between B12DA and autoimmune hypothyroidism (the clinical phenotype), as well as between B12DA and Hashimoto thyroiditis (the underlying autoimmune etiology). Our findings indicate that both B12DA and Hashimoto thyroiditis are risk factors for hypothyroidism, and that B12DA is a risk factor for Hashimoto thyroiditis and autoimmune hypothyroidism. MVMR analysis further revealed that, even after adjusting for each factor, the causal relationships among B12DA, Hashimoto thyroiditis, and hypothyroidism persisted. Moreover, our findings indicate that Hashimoto thyroiditis and B12DA are not only associated with hypothyroidism but are also causally linked with thyrotoxicosis, which is consistent with the clinical features of Hashimoto thyroiditis that often leads to hypothyroidism and, occasionally, transient thyrotoxicosis.^[23]^ MVMR analysis further revealed that having adjusted for Hashimoto thyroiditis, we no longer detected a causal relationship between B12DA and thyrotoxicosis. In summary, Hashimoto thyroiditis appears to partially mediate the causal effect of B12DA on hypothyroidism, whereas the causal effect of B12DA on thyrotoxicosis appears to be fully mediated by Hashimoto thyroiditis. This causal relationship between B12DA and hypothyroidism, along with the mediating role of Hashimoto thyroiditis, further emphasizes the complex and multifaceted nature of thyroid autoimmunity and its effects on thyroid hormone levels. The fact that our findings indicating Hashimoto thyroiditis to be causally associated with hypothyroidism and thyrotoxicosis is consistent with current clinical understanding lends credibility to the approach adopted in this study.

Although we established a causal relationship between B12DA and hypothyroidism, the details of the pathway by which B12DA contributes to the development of hypothyroidism remain unclear. Our analysis of genetic variants associated with B12DA and hypothyroidism revealed potential associations with autoimmune diseases, and our MR analyses of B12DA and autoimmune hypothyroidism confirmed this association. Autoimmune diseases are complex disorders that often affect multiple organs and frequently co-occur, making it challenging to establish a singular connection between B12DA and specific autoimmune conditions. To the best of our knowledge, this study is the first in which MVMR has been used to demonstrate that B12DA leads to hypothyroidism with Hashimoto thyroiditis playing a mediating role, thereby serving to highlight the complexity of these disorders and emphasizing the novelty and significance of our findings.

Nutrients are essential for maintaining adequate physical health, with a balanced diet providing the essential vitamins, minerals, proteins, fats, and carbohydrates that collectively contribute to preventing a wide range of diseases, including cancer,^[24]^ cardiovascular conditions, and diabetes.^[25]^ In addition, Mikulska et al have emphasized the pivotal role of appropriate medical therapy, along with dietary modifications and supplementation, as fundamental elements of healthcare for patients with Hashimoto thyroiditis. These authors systematically reviewed the effects of different nutrients associated with Hashimoto thyroiditis, including selenium, iodine, and vitamin D, on treatment outcomes.^[26]^ Our findings in this study corroborate the genetic association between B12DA and hypothyroidism, as well as Hashimoto thyroiditis, which thus provides evidence to indicate that vitamin B_12_ should be an integral component of nutritional intervention strategies for hypothyroidism and Hashimoto thyroiditis, in that it may potentially address the underlying genetic factors contributing to these conditions.

Although significant heterogeneity was observed in the MR analysis of the association between B12DA and hypothyroidism (Cochran *Q* test, *P* < .05), the MR-Egger intercept test did not suggest the presence of horizontal pleiotropy (*P* > .05). In addition, the leave-one-out sensitivity analysis (see Figure S1) showed that the causal estimates remained stable after the sequential removal of each instrumental variable. These findings indicate that the selected instrumental variables are less likely to affect the outcome through alternative biological pathways bypassing the exposure, and that the causal estimates derived from the primary analysis (IVW method) are unlikely to be biased by pleiotropy, supporting the robustness of the overall causal inference.^[9,10]^ Moreover, although autoimmune-related pleiotropy and phenotypic heterogeneity may partly contribute to the observed variability, they are unlikely to be the predominant drivers of the observed heterogeneity. Notably, the positive causal association persisted after exclusion of the autoimmune thyroid disease-related variant rs1990760, suggesting that the heterogeneity was unlikely to be primarily driven by strong directional pleiotropy or by the effect of a single autoimmune-related locus.

Specifically, heterogeneity may arise from several sources.^[3]^ First, with respect to population stratification, genetic associations may differ across ethnic groups or subpopulations, potentially biasing the causal estimates.^[27]^ Second, phenotypic heterogeneity represents another important source.^[28]^ The genetic instruments for B12DA used in this study are enriched in autoimmune-related genomic regions; however, the GWAS data for hypothyroidism encompass multiple subtypes, including autoimmune and nonautoimmune causes. Phenotypic heterogeneity in autoimmune diseases may therefore contribute to variability in the effect estimates. Notably, significant heterogeneity persisted in our subgroup MR analysis focusing on hypothyroidism due to autoimmune thyroid disease, suggesting that factors beyond phenotypic heterogeneity of the outcome also play a role. Finally, a nonlinear relationship between B12DA and hypothyroidism may also contribute to heterogeneity, meaning that the effect of genetic instruments on the outcome may differ across different exposure levels.^[29]^ The findings of other studies, indicating that changes in Hb concentration in patients with thyroid dysfunction are clinically irrelevant,^[30]^ may also explain this phenomenon. In MR analysis of the causal relationship between Hashimoto thyroiditis and hypothyroidism, results from the MR-Egger intercept test provided evidence of horizontal pleiotropy, on the basis of which we scrutinized the outcomes of 4 additional MR techniques to ensure the accuracy of our causal effect estimates. This approach enabled us to circumvent the influence of horizontal pleiotropy, whereby a single genetic variant influences multiple traits, and strengthened the robustness of our causal inferences.^[31,32]^

Nevertheless, although our results are encouraging, the study does have certain limitations. Notably, the sample size may not have been sufficiently large to detect more subtle effects; for example, the influence of other forms of nutritional anemia, such as the anemia caused by folic acid deficiency and its effect on hyperthyroidism. Additionally, the study was restricted to European populations, thereby limiting the generalizability of our findings. To address these issues, we used multiple databases, which increased the overall sample size and enhanced the statistical power of our study. However, limited data availability poses challenges in analyzing the causal relationship between B12DA and autoimmune hypothyroidism and evaluating the mediating role in 2-step MR analyses. Using the same database for both exposure and outcome data may lead to sample overlap, which could influence the reliability of causal inferences, although this was not the main finding of this study. Moreover, the causal association between B12DA and autoimmune hypothyroidism identified in this study has implications for clinical treatment. However, further research is needed in this regard. Furthermore, we used MR analysis to assess the lifetime impact of anemia on hypothyroidism, providing a time- and cost-effective alternative to long-term randomized controlled trials. However, this approach has limitations, including the lack of assessment of the effects at different stages of anemia. Although MR can contribute to determining the direction of causation, it does not completely eliminate the possibility of reverse causation, particularly if the disease process is bidirectional. Finally, we did not seek to assess the effects of preanemia (characterized only by vitamin B_12_ deficiency), or the effects of varying degrees of deficiency on thyroid function. Consequently, long-term randomized controlled trials are necessary to study and analyze the different developmental stages of development of anemia and hypothyroidism and identify measurable indicators.

## 5. Conclusion

This is the 1st study to provide evidence of a significant causal relationship between B12DA and hypothyroidism. Our study showed that after adjusting for IDA and Hashimoto thyroiditis, B12DA had a significant causal effect on hypothyroidism. The study further indicated that Hashimoto thyroiditis plays a partial mediating role in this causal effect. Importantly, the genetic evidence suggests a possible shared autoimmune susceptibility linking B12DA, Hashimoto thyroiditis, and hypothyroidism, rather than a straightforward nutrient-deficiency-driven pathway. Therefore, while our findings highlight a statistical causal association, they should not be overinterpreted as evidence that treating B12DA alone will directly reduce the risk of autoimmune thyroid disease. Instead, the observed link may reflect underlying immune dysregulation. Clinically, screening for B12DA in patients with hypothyroidism or Hashimoto thyroiditis is warranted, and vice versa. However, nutrient supplementation should be considered as part of a broader clinical evaluation that includes assessment of autoimmune status. Future interventional and mechanistic studies are needed before recommending B12DA treatment as a preventive strategy for hypothyroidism.

## Acknowledgments

This study used data from publicly accessible GWAS datasets. The authors are grateful to all investigators and participants who contributed to the original studies and data collection. This research was funded by the Quanzhou City Science and Technology Program of China (Grant No. 2020N057s). The funder had no role in the study design, data analysis, interpretation of the results, decision to publish, or preparation of the manuscript.

## Author contributions

**Conceptualization:** Liyun Chen, Xiali Wang

**Data curation:** Liyun Chen, Sijing Huang, Jinfen Kang

**Formal analysis:** Liyun Chen, Wensen Huang, Jinyan Zhang

**Funding acquisition:** Xiali Wang

**Methodology:** Liyun Chen

**Project administration:** Liyun Chen

**Software:** Liyun Chen

**Supervision:** Wensen Huang

**Validation:** Liyun Chen, Xiali Wang

**Visualization:** Qingyi Wu

**Writing – original draft:** Liyun Chen

**Writing – review & editing:** Xiali Wang





**Figure:**