# Supplementary material for: Unveiling the clinical connections between vitamin B12 deficiency anemia, Hashimoto thyroiditis, and hypothyroidism: New insights from Mendelian randomization studies
Source: Medicine (Baltimore). 2026 May 15;105(20):e48901. doi: 10.1097/MD.0000000000048901 (PMC13183166; doi:10.1097/MD.0000000000048901)
Supplement: Supplementary file 1 [file medi-105-e48901-s001.docx]

**Supplementary TableS1.** Results of univariate Mendelian randomization analysis

| **exposure** | **outcome** | **method** | **nsnp** | **b** | **se** | **pval** | **OR（95%CI）** |
| --- | --- | --- | --- | --- | --- | --- | --- |
| IDA* | hypothyroidism | IVW | 2 | 0.132 | 0.045 | 3.44E-03 | 1.14(1.04-1.25) |
| B12DA* |  | MR Egger | 4 | 1.339 | 0.275 | 3.97E-02 | 3.81(2.23-6.54) |
|  |  | WME | 4 | 0.265 | 0.057 | 3.75E-06 | 1.30(1.17-1.46) |
|  |  | IVW | 4 | 0.404 | 0.192 | 3.55E-02 | 1.50(1.03-2.18) |
|  |  | SM | 4 | 0.427 | 0.088 | 1.69E-02 | 1.53(1.29-1.82) |
|  |  | WM | 4 | 0.343 | 0.123 | 6.84E-02 | 1.41(1.11-1.79) |
| other nutritional naemias |  | MR Egger | 7 | 0.004 | 0.005 | 4.63E-01 | 1.00(0.99-1.01) |
|  |  | WME | 7 | 0.001 | 0.005 | 7.92E-01 | 1.00(0.99-1.01) |
|  |  | IVW | 7 | 0.002 | 0.004 | 5.37E-01 | 1.00(0.99-1.01) |
|  |  | SM | 7 | -0.001 | 0.009 | 8.89E-01 | 1.00(0.98-1.02) |
|  |  | WM | 7 | -0.002 | 0.007 | 7.75E-01 | 1.00(0.98-1.01) |
| Hashimoto  thyroiditis* |  | MR Egger | 13 | 1.132 | 0.057 | 5.35E-10 | 3.10(2.78-3.46) |
|  |  | WME | 13 | 1.001 | 0.050 | 1.29E-88 | 2.72(2.47-3.00) |
|  |  | IVW | 13 | 1.010 | 0.025 | 0 | 2.74(2.61-2.88) |
|  |  | SM | 13 | 1.003 | 0.077 | 1.90E-08 | 2.73(2.35-3.17) |
|  |  | WM | 13 | 0.993 | 0.067 | 4.65E-09 | 2.70(2.37-3.08) |
| B12DA | autoimmune  hypothyroidism* | MR Egger | 5 | 0.930 | 0.199 | 1.86E-02 | 2.54(1.72-3.75) |
|  |  | WME | 5 | 0.361 | 0.053 | 1.03E-11 | 1.44(1.29-1.59) |
|  |  | IVW | 5 | 0.491 | 0.099 | 6.07E-07 | 1.63(1.35-1.98) |
|  |  | SM | 5 | 0.411 | 0.135 | 3.87E-02 | 1.51(1.16-1.97) |
|  |  | WM | 5 | 0.237 | 0.073 | 3.18E-02 | 1.27(1.10-1.46) |
|  | Hashimoto  thyroiditis* | MR Egger | 5 | 0.949 | 0.222 | 2.35E-02 | 2.58(1.67-3.99) |
|  |  | WME | 5 | 0.324 | 0.066 | 1.00E-06 | 1.38(1.21-1.58) |
|  |  | IVW | 5 | 0.405 | 0.119 | 6.75E-04 | 1.50(1.19-1.89) |
|  |  | SM | 5 | 0.318 | 0.112 | 4.68E-02 | 1.37(1.10-1.71) |

**Supplementary Table S1 Continued**

| **exposure** | **outcome** | **method** | **nsnp** | **b** | **se** | **pval** | **OR（95%CI）** |
| --- | --- | --- | --- | --- | --- | --- | --- |
| B12DA | Hashimoto  thyroiditis* | WM | 5 | 0.327 | 0.147 | 9.08E-02 | 1.39(1.04-1.85) |
| B12DA* | thyrotoxicosis | MR Egger | 4 | 0.006 | 0.001 | 5.19E-02 | 1.01(1.00-1.01) |
|  |  | WME | 4 | 0.003 | 0.001 | 1.92E-05 | 1.00(1.00-1.00) |
|  |  | IVW | 4 | 0.004 | 0.001 | 5.59E-09 | 1.00(1.00-1.01) |
|  |  | SM | 4 | 0.003 | 0.001 | 7.22E-02 | 1.00(1.00-1.00) |
|  |  | WM | 4 | 0.003 | 0.001 | 9.42E-02 | 1.00(1.00-1.01) |
| Hashimoto  thyroiditis* |  | MR Egger | 10 | 0.010 | 0.004 | 2.90E-02 | 1.01(1.00-1.02) |
|  |  | WME | 10 | 0.006 | 0.001 | 1.43E-11 | 1.01(1.00-1.01) |
|  |  | IVW | 10 | 0.006 | 0.001 | 4.06E-06 | 1.01(1.00-1.01) |
|  |  | SM | 10 | 0.006 | 0.002 | 6.71E-03 | 1.01(1.00-1.01) |
|  |  | WM | 10 | 0.007 | 0.001 | 1.70E-04 | 1.01(1.00-1.01) |
| hypothyroidism | IDA* | MR Egger | 68 | 0.088 | 0.054 | 1.12E-01 | 1.09(0.98-1.21) |
|  |  | WME | 68 | 0.113 | 0.039 | 4.10E-03 | 1.12(1.04-1.21) |
|  |  | IVW | 68 | 0.096 | 0.025 | 1.49E-04 | 1.10(1.05-1.16) |
|  |  | SM | 68 | 0.096 | 0.076 | 2.11E-01 | 1.10(0.95-1.28) |
|  |  | WM | 68 | 0.107 | 0.047 | 2.57E-02 | 1.11(1.02-1.22) |
|  | B12DA* | MR Egger | 68 | 0.638 | 0.187 | 1.08E-03 | 1.89(1.31-2.73) |
|  |  | WME | 68 | 0.568 | 0.094 | 1.29E-09 | 1.76(1.47-2.12) |
|  |  | IVW | 68 | 0.601 | 0.086 | 2.28E-12 | 1.82(1.54-2.16) |
|  |  | SM | 68 | 0.847 | 0.289 | 4.65E-03 | 2.33(1.32-4.11) |
|  |  | WM | 68 | 0.246 | 0.244 | 3.19E-01 | 1.28(0.79-2.06) |
| thyrotoxicosis | B12DA | MR Egger | 4 | 327.712 | 387.773 | 4.87E-01 | 2.11E+142(0.00-Inf) |
|  |  | WME | 4 | 17.757 | 12.877 | 1.68E-01 | 5.15E+07(0.00-4.71E+18) |

**Supplementary Table S1 Continued**

| **exposure** | **outcome** | **method** | **nsnp** | **b** | **se** | **pval** | **OR（95%CI）** |
| --- | --- | --- | --- | --- | --- | --- | --- |
| thyrotoxicosis | B12DA | IVW | 4 | 50.402 | 53.235 | 3.44E-01 | 7.75E+21(0.00-1.60E+67) |
|  |  | SM | 4 | -3.487 | 21.236 | 8.80E-01 | 3.000000E-02(0.00-3.650462E+16) |
|  |  | MR Egger | 4 | 327.712 | 387.773 | 4.87E-01 | 2.11E+142(0.00-Inf) |
| Hashimoto  thyroiditis* |  | MR Egger | 12 | 0.953 | 0.502 | 8.67E-02 | 2.59(0.97-6.93) |
|  |  | WME | 12 | 0.371 | 0.123 | 2.47E-03 | 1.45(1.14-1.84) |
|  |  | IVW | 12 | 0.604 | 0.174 | 5.02E-04 | 1.83(1.30-2.57) |
|  |  | SM | 12 | 0.424 | 0.278 | 1.55E-01 | 1.53(0.89-2.64) |
|  |  | WM | 12 | 0.279 | 0.191 | 1.71E-01 | 1.32(0.91-1.92) |

**P*＜0.05, IDA iron deficiency anemia, B12DA vitamin B12 deficiency anemia, nsnp nonsynonymous single nucleotide polymorphism, se standard error, pval probability value, OR odds ratio, CI confidence interval, WME weighted median estimator, IVW Inverse variance weighted, SM Simple Mode, WM Weighted Mode.
