# Supplementary material for: Unveiling the clinical connections between vitamin B12 deficiency anemia, Hashimoto thyroiditis, and hypothyroidism: New insights from Mendelian randomization studies
Source: Medicine (Baltimore). 2026 May 15;105(20):e48901. doi: 10.1097/MD.0000000000048901 (PMC13183166; doi:10.1097/MD.0000000000048901)
Supplement: Supplementary file 2 [file medi-105-e48901-s003.docx]

**Supplementary Table S2**. Result of heterogeneity and horizontal pleiotropy

| **exposure** | **outcome** | **Cochran Q Test** | | **MR-Egger intercept test** | |
| --- | --- | --- | --- | --- | --- |
|  |  | **Q** | ***P*** | **Intercept** | ***P*** |
| IDA | hypothyroidism | 0.227 | 0.634 | - | - |
| B12DA* |  | 277.064 | 9.57E-59* | -0.221 | 0.107 |
| other nutritional  naemias |  | 4.80 | 0.569 | -0.0037 | 0.638 |
| hypothyroidism | IDA | 59.050 | 0.745 | 0.001 | 0.857 |
|  | B12DA* | 215.511 | 1.71E-17* | -0.004 | 0.825 |
| B12DA | autoimmune hypothyroidism* | 89.694 | 1.53E-18* | -0.141 | 0.101 |
|  | Hashimoto thyroiditis* | 63.842 | 4.51E-13* | -0.176 | 0.08 |
|  | thyrotoxicosis | 6.743 | 0.081 | -0.001 | 0.213 |
| Hashimoto thyroiditis | hypothyroidism* | 20.058 | 0.066 | -0.022 | 0.040* |
|  | thyrotoxicosis* | 97.288 | 5.56E-17* | -6.68E-04 | 0.332 |
| thyrotoxicosis* | B12DA | 90.668 | 1.57E-19* | -0.631 | 0.545 |
| Hashimoto thyroiditis* |  | 75.209 | 1.24E-11* | -0.0645 | 0.474 |

******P*＜0.05, IDA iron deficiency anemia, B12DA vitamin B12 deficiency anemia, *P* probability value.
